# Associations of preoperative anaemia with healthcare resource use and outcomes after colorectal surgery: a population-based cohort study

**DOI:** 10.1016/j.bja.2024.03.018

**Published:** 2024-04-20

**Authors:** Lily J. Park, Husein Moloo, Tim Ramsay, Kednapa Thavorn, Justin Presseau, Terry Zwiep, Guillaume Martel, P.J. Devereaux, Robert Talarico, Daniel I. McIsaac

**Affiliations:** 1 Population Health Research Institute, Hamilton, ON, Canada; 2 Department of Health Research Methods, Evidence & Impact, McMaster University, Hamilton, ON, Canada; 3 Department of Surgery, Division of General Surgery, McMaster University, Hamilton, ON, Canada; 4 Ottawa Hospital Research Institute, Ottawa Hospital, Ottawa, ON, Canada; 5 Department of Surgery, Division of Colorectal Surgery, Ottawa, ON, Canada; 6 School of Epidemiology and Public Health, University of Ottawa, Ottawa, ON, Canada; 7 ICES, Toronto, ON, Canada; 8 Department of Surgery, Division of General Surgery, University of Western Ontario, London, ON, Canada; 9 Department of Surgery, Division of Hepatobiliary Surgery, University of Ottawa, Ottawa, ON, Canada; 10 Department of Medicine, Division of Cardiology, McMaster University, Hamilton, ON, Canada; 11 Departments of Anesthesiology and Pain Medicine, The Ottawa Hospital and the University of Ottawa, Ottawa, ON, Canada

**Keywords:** anaemia, colorectal surgery, perioperative care, surgery, transfusion

## Abstract

**Background:**

Preoperative anaemia is common in patient undergoing colorectal surgery. Understanding the population-level costs of preoperative anaemia will inform development and evaluation of anaemia management at health system levels.

**Methods:**

This was a population-based cohort study using linked, routinely collected data, including residents from Ontario, Canada, aged ≥18 yr who underwent an elective colorectal resection between 2012 and 2022. Primary exposure was preoperative anaemia (haemoglobin <130 g L^−1^ in males; <120 g L^−1^ in females). Primary outcome was 30-day costs in 2022 Canadian dollars (CAD), from the perspective of a publicly funded healthcare system. Secondary outcomes included red blood cell transfusion, major adverse events (MAEs), length of stay (LOS), days alive at home (DAH), and readmissions.

**Results:**

We included 54,286 patients, with mean 65.3 (range 18–102) years of age and 49.0% females, among which 21 264 (39.2%) had preoperative anaemia. There was an absolute adjusted cost increase of $2671 per person at 30 days after surgery attributable to preoperative anaemia (ratio of means [RoM] 1.05, 95% confidence interval [CI] 1.04–1.06). Compared with the control group, 30-day risks of transfusion (odds ratio [OR] 4.34, 95% CI 4.04–4.66), MAEs (OR 1.14, 95% CI 1.03–1.27), LOS (RoM 1.08, 95% CI 1.07–1.10), and readmissions (OR 1.16, 95% CI 1.08–1.24) were higher in the anaemia group, with reduced DAH (RoM 0.95, 95% CI 0.95–0.96).

**Conclusions:**

Approximately $2671 CAD per person in 30-day health system costs are attributable to preoperative anaemia after colorectal surgery in Ontario, Canada.

**Editor's key points**

• Anaemia is now widely recognised to be an important risk factor for poor perioperative outcomes.
• However, it is unclear whether correcting anaemia before, during, and after surgery reduces the risk of poor outcomes.
• The evidence base for interventions such as blood transfusion and i.v. iron is complex and does not present a clear case for routine perioperative correction of anaemia.
• The current study estimates the additional healthcare costs of perioperative treatments required for treatment of preoperative anaemia.
• These findings suggest that, if proven clinically effective, treating preoperative anaemia would also be cost-effective in the perioperative period.

Preoperative anaemia is present in more than 40% of patients undergoing colorectal surgery, and is known to be associated with poor outcomes, including higher rates of mortality, complications, length of stay (LOS), and readmissions.^1^, ^2^, ^3^, ^4^, ^5^, ^6^ A study using National Surgical Quality Improvement Program data demonstrated an increased associated risk of acute renal failure, stroke, myocardial infarction, and death (odds ratio [OR] 1.49–2.19), with a dose–response relationship between anaemia severity and these outcomes.^1^^,^^7^^,^^8^

Despite this growing evidence, multifactorial barriers to preoperative anaemia optimisation exist. First, healthcare providers are not always aware of the importance of preoperative anaemia and its association with poor outcomes.^2^^,^^9^ Additionally, perioperative care, including anaemia optimisation, requires collaborative efforts from various practitioners such as primary care physicians, anaesthesiologists, perioperative care physicians, and surgeons. The lack of clearly defined roles among the patient's circle of care in managing and monitoring anaemia may often lead to its oversight.^10^^,^^11^ Data supporting the effectiveness of preoperative anaemia management are also uncertain. For example, a multicentre trial involving 487 participants did not find evidence of reduced red blood cell (RBC) transfusion with administration of i.v. iron therapy in anaemic abdominal surgery patients (risk ratio [RR] 0.98, 95% confidence interval [CI] 0.68–1.43).^12^ However, this trial was underpowered, may have lacked ideal alignment of its intervention to anaemia aetiology in all participants, and faced challenges in timely delivery of iron therapy. Thus, these data do not preclude the possibility that i.v. iron therapy and other interventions aimed at anaemia management could provide benefit. Finally, the paucity of data on the economic impact of preoperative anaemia may also be a barrier to development of effective management strategies.^2^^,^^13^

Understanding the resource use and outcome burden attributable to anaemia at a population level could help prioritise development of effective anaemia management strategies and inform economic evaluation of perioperative blood management (PBM) programmes. We conducted a population-based cohort study to estimate the association of preoperative anaemia with health system costs and clinical outcomes in adult patients undergoing elective colorectal surgery.

## Methods

### Design and setting

After protocol registration (https://doi.org/10.17605/OSF.IO/A586T), we conducted a population-based, retrospective cohort study in Ontario, Canada, where all residents have access to healthcare through the provincial health insurance. In Ontario, standardised methods are used to collect and store linked healthcare data at ICES (formerly known as the Institute for Clinical Evaluative Sciences). The dataset was prepared and analysed by a trained data analyst and is reported following recommended methods (Supplementary Appendix I).^14^^,^^15^ The study was legally waived from research ethics review per provincial health privacy legislation.

### Cohort

Ontario residents aged ≥18 yr who underwent elective colorectal resection between April 1, 2012 and March 31, 2022 were identified for inclusion. The Canadian Classification of Intervention codes for colon and rectal resections were used to identify eligible patients (Supplementary Appendix II), which have demonstrated validity.^16^ The cohort was limited to 1) elective surgeries only, as emergency patients would not have time for optimisation of haemoglobin concentrations before surgery and 2) an individual's first surgery if they received multiple surgeries within the study period.

### Exposure

The primary exposure was anaemia as defined by the World Health Organization's (WHO) guidelines (Hgb <130 g L^−1^ in males, <120 g L^−1^ in females).^17^ We evaluated the haemoglobin value most proximal to surgery between 90 days and 1 day before surgery to avoid misclassification of an intraoperative or postoperative value. Secondary representations of haemoglobin (that were not sex dependent) included a continuous linear, restricted cubic spline to allow for a continuous, but potentially nonlinear association, and categorical representation of WHO severity (not anaemic, mild [110 g L^−1^ normal], moderate [80 to <110 g L^−1^], and severe [<80 g L^−1^]).^17^

### Outcome

The primary outcome was total healthcare costs at 30 days (i.e. costs starting from the day of surgery to postoperative day 29) from the perspective of the universal public healthcare system. Costs were normalised to 2022 Canadian dollars (CAD) and were calculated using validated patient-level costing algorithms that include direct and non-direct medical costs associated with all patient contacts with the healthcare system (Supplementary Appendix III).^18^ This method does not account for production costs of RBC units, which are paid for by Canadian Blood Services, and out-of-pocket patient expenses.

Secondary outcomes included health system costs in the 90 and 365 days after surgery, in-hospital major adverse events [MAEs] (composite of major cardiac and renal complications or death), LOS, receipt of any RBC transfusion, 30-day readmissions, and days alive at home. Specific details and diagnostic codes are listed in Supplementary Appendix IV.

### Confounders

Potential confounders were selected based on clinical and epidemiological knowledge of factors that we postulated would be associated with anaemia and dependent variables (Supplementary Appendix V). These included patient characteristics, Elixhauser comorbidities, frailty index score, total health system costs accrued in the year before surgery, the specific surgical procedure, receipt of chemotherapy in the 6 months before surgery, estimated glomerular filtration rate, presence of a cancer tissue diagnoses of colon or rectal cancer before surgery, and presence of inflammatory bowel disease.^19^^,^^20^

### Sample size

As a population-based study, we planned to include all patients who underwent colorectal surgery from 2012 to 2022. With an estimate that at least 4000 colorectal procedures are performed annually in Ontario and existing data demonstrating mean case costs of $20,000 CAD (standard deviation [sd] $23,000), we determined that we would require a minimum of 1119 participants for a linear regression model with an explained variance of 0.2.^21^, ^22^, ^23^, ^24^

### Missing data

As 22.3% of exposure data were missing, we followed our pre-specified approach using multiple imputation to create 25 complete datasets based on Rubin's rules.^25^ Predictive mean matching via multivariate chained equations (MICE) was used to impute missing haemoglobin values directly, under a missing at random assumption. There were no complete gaps in haemoglobin testing across time or centre. However, there were greater percentage of missing data in the earlier years of the observation period. For instance, 26.1% of the missing haemoglobin values were from 2012, 19.7% from 2013, 14.5% from 2014, 10.6% from 2015, with percentages of missing data from each year decreasing thereafter. This might be owing to increasing awareness for the importance of preoperative anaemia detection. Further information on the diagnostics for multiple imputation can be found in Supplementary Appendix VIa–c.

### Data analysis

Descriptive statistics were calculated comparing characteristics between people with and without preoperative anaemia. Standardised differences were calculated to identify differences that may be substantive (i.e. standardised difference >0.10).^26^ Unadjusted and adjusted outcome differences between exposure levels were analysed using generalised linear models. In addition to the exposure variable, all adjusted models included control for pre-specified confounders while accounting for clustering by hospital using generalised estimating equations. Cost and LOS were modelled using log-transformed values in a linear model; binary outcomes were modelled using logistic models; days alive at home was modelled using the negative-binomial regression. For primary cost outcomes, the adjusted model's predicted values were used to generate adjusted absolute differences and CIs computed via bootstrapped standard errors of 200 replicates.^27^

### Sensitivity analysis

Pre-specified sensitivity analyses were conducted using representations of anaemia and haemoglobin concentration that were not sex dependent. Specifically, the primary analysis was re-run with a continuous, then a continuous nonlinear (restricted cubic spline) of haemoglobin concentration, then the four-level WHO anaemia severity categorisation as exposure variables. *Post hoc*, we re-ran the primary model with days alive during the 30-day window as an offset to account for the impact of early mortality on cost accumulation, and an analysis that accounted for estimated transfusion costs (as our data did not capture the costs of RBC units transfused (Supplementary Appendix VII).

### Effect modification

Effect modifier analyses using multiplicative interaction terms between binary exposure and the following factors were conducted: oncologic *vs* benign surgical indication; cancer stage (limited to patients with colorectal cancer); frailty index score; age; sex; neighbourhood income quintiles. We also conducted effect modifier analyses using interaction terms of continuous haemoglobin with sex and age. *P*-values <0.05 were considered significant for interaction.

## Results

**Fig 1:**
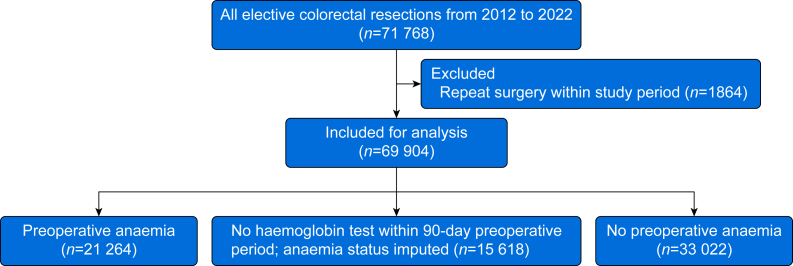
STROBE flow diagram for patient inclusion.

Figure 1 demonstrates the patient flow diagram for inclusion and analysis. Overall, 69,904 patients undergoing elective colorectal surgery in Ontario between 2012 and 2022 were included; 54,286 (77.7%) had complete exposure data. Among these, 21,264 (39.2%) had preoperative anaemia, 35,108 (64.7%) patients had a colorectal cancer diagnosis, and 2167 (4.0%) patients had inflammatory bowel disease. Patients with anaemia were older, lived with greater frailty, tended to have more comorbidities, and were more likely to have a cancer indication for surgery than those without anaemia (Table 1). Baseline characteristics of the study population with and without complete exposure data can be seen in Supplementary Table S1.

**Table 1:** Baseline characteristics of study population. eGFR, estimated glomerular filtration rate. ∗∗absolute standardized differences >0.1 represent a substantial difference.

Patient characteristics	Non-anaemic *N*=33 022	Anaemic *N*=21 264	Absolute standardised difference∗∗
Age, median (range), years	63 (18–98)	70 (18–102)	0.51
Female, *n* (%)	16**,**043 (48.6)	10**,**530 (49.5)	0.02
Rural, *n* (%)	4884 (14.8)	2968 (14.0)	0.02
**Neighbourhood income quintile, *n* (%)**			
1 (highest)	5935 (18.0)	4452 (20.9)	0.07
2	6456 (19.6)	4458 (21.0)	0.04
3	6661 (20.2)	4195 (19.7)	0.01
4	6899 (20.9)	4078 (19.2)	0.04
5 (lowest)	7001 (21.2)	4017 (18.9)	0.06
Academic hospital (*vs* community), *n* (%)	10**,**900 (33.0)	6937 (32.6)	0.01
**Baseline health status**			
Frailty index, mean (sd)	0.10 (0.06)	0.15 (0.08)	0.66
Haemoglobin value, g L^-1^, mean (sd)	140 (11)	106 (14)	2.66
Days from haemoglobin test to surgery, mean (sd)	18 (20)	18 (20)	0
eGFR, mean (sd)	83.05 (17.93)	73.43 (23.76)	0.46
Inflammatory bowel disease, *n* (%)	1311 (4.0)	856 (4.0)	0
**Elixhauser comorbidities, *n* (%)**			
Diabetes mellitus complicated	1742 (5.3)	3064 (14.4)	0.31
Diabetes mellitus uncomplicated	4429 (13.4)	4541 (21.4)	0.21
Congestive heart failure	540 (1.6)	1306 (6.1)	0.23
Hypertension uncomplicated	6096 (18.5)	6335 (29.8)	0.27
Hypertension complicated	35 (0.1)	79 (0.4)	0.05
Chronic pulmonary disease	1156 (3.5)	1243 (5.8)	0.11
Dementia	74 (0.2)	216 (1.0)	0.1
Cerebrovascular disease	290 (0.9)	457 (2.1)	0.1
Chronic renal disease	149 (0.5)	499 (2.3)	0.16
Dialysis	38 (0.1)	176 (0.8)	0.1
Cancer	21,413 (64.8)	17,937 (84.4)	0.46
Cancer with metastases	4527 (13.7)	4629 (21.8)	0.21
Peripheral vascular disease	311 (0.9)	487 (2.3)	0.11
Liver disease	219 (0.7)	243 (1.1)	0.05
Peptic ulcer disease	319 (1.0)	589 (2.8)	0.13
Rheumatic disease	87 (0.3)	106 (0.5)	0.04
Hemiparesis or hemiplegia	35 (0.1)	89 (0.4)	0.06
Atrial fibrillation	707 (2.1)	1332 (6.3)	0.21
Venous thromboembolism	134 (0.4)	191 (0.9)	0.06
Cardiac valve disease	243 (0.7)	524 (2.5)	0.14
Disease of the pulmonary circulation	361 (1.1)	525 (2.5%)	0.1
Coagulopathy	213 (0.6)	371 (1.7)	0.1
Obesity	1104 (3.3)	692 (3.3)	0
Weight loss	561 (1.7)	752 (3.5)	0.12
Blood loss anaemia	1792 (5.4)	2478 (11.7)	0.22
Nutritional deficiency anaemia	73 (0.2)	334 (1.6)	0.14
Alcohol abuse	293 (0.9)	268 (1.3)	0.04
Drug abuse	84 (0.3)	67 (0.3)	0.01
Psychoses	52 (0.2)	64 (0.3)	0.03
Depression	379 (1.1)	343 (1.6)	0.04
**Baseline cancer status, *n* (%)**			
Cancer diagnosis	19,098 (57.8)	16,010 (75.3)	0.38
Colon cancer	9864 (29.9)	11,183 (52.6)	0.47
Rectal cancer	6910 (20.9)	3308 (15.6)	0.14
**Cancer stage (among cancer patients), *n* (%)**	20,131 (61.0)	9915 (46.6)	0.29
0	77 (0.2)	15 (0.1)	0.04
I	4394 (13.3)	2104 (9.9)	0.11
II	3397 (10.3)	4381 (20.6)	0.29
III	4272 (12.9)	3937 (18.5)	0.15
IV	751 (2.3)	912 (4.3)	0.11
Previous chemotherapy	3294 (10.0)	2641 (12.4)	0.08
**Surgery type, *n* (%)**			
Partial colectomy	19,722 (59.7)	15,413 (72.5)	0.27
Subtotal colectomy	327 (1.0)	261 (1.2)	0.02
Proctocolectomy	129 (0.4)	132 (0.6)	0.03
Low anterior resection	9591 (29.0)	3786 (17.8)	0.27
Abdominoperineal resection	3253 (9.9)	1672 (7.9)	0.07

### Attributable cost of anaemia

The unadjusted mean 30-day total healthcare costs among those with and without anaemia was $22,810 (sd $16,495) and $19,274 (sd $12,071), respectively. The unadjusted ratio of means (RoM) for costs at 30 days in patients with anaemia compared with those without anaemia was 1.14 (95% CI 1.13–1.15). After multivariable adjustment accounting for hospital clustering, we estimated a significant relative increase in mean costs for those with anaemia (adjusted RoM [aROM] of 1.05 (95% CI 1.04–1.06). The absolute adjusted cost attributable to anaemia was estimated to be $2671 (95% CI $2661–2680). Costs were also higher for those with preoperative anaemia across the first year after surgery (90-day aRoM 1.10, 95% CI 1.08–1.11; 365-day aRoM 1.16, 95% CI 1.15–1.18). The mean absolute adjusted cost differences at 90 and 365 days after surgery were $4748 (95% CI $4734–$4762) and $10,435 (95% CI $10,406–$10,464), respectively. Cost data are provided in Table 2.

**Table 2:** Costs associated with anaemia status. SD, standard deviation; RBC, red blood cell. ∗All costs are in 2022 adjusted Canadian dollars; ^†^Adjusted covariates: age, sex, neighbourhood income quintile, rural *vs* urban residence, Elixhauser comorbidities, presence of colon or rectal cancer tissue diagnosis, frailty index score, preoperative healthcare resource utilisation, the specific surgical procedure, receipt of chemotherapy in the 6 months before surgery, estimated glomerular filtration rate, and presence of inflammatory bowel disease.

Postoperative day	Mean (sd) health system costs∗		Ratio of means	
	Not anaemic	Anaemic	Unadjusted	Adjusted^†^
**30**	19,274 (12,071)	22,810 (16,495)	1.14 (1.13–1.15)	1.05 (1.04–1.06)
**90**	24,588 (19,955)	30,943 (28,040)	1.21 (1.19–1.22)	1.10 (1.08–1.11)
**365**	39,031 (37,744)	52,184 (51,149)	1.32 (1.30–1.34)	1.16 (1.15–1.18)
**30 (including RBC unit**	19,345 (12,173)	23,106 (16,675)	1.15 (1.14–1.17)	1.06 (1.05–1.07)

### Sensitivity analyses

**Fig 2:**
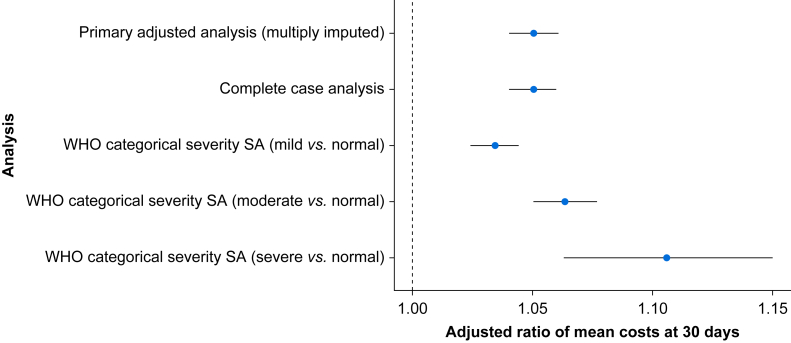
Forest plot comparing primary analysis with sensitivity analysis (SA) estimates of binary and categorical exposures. For each association, the figure reports the ratio of means (large circle) and 95% confidence interval comparing mean costs between those with *vs* without anaemia (unless otherwise indicated).

Complete case analyses were consistent with the primary analysis. All parameterisations of anaemia demonstrated significantly increased adjusted costs for patients with anaemia, lower haemoglobin concentrations, or both. When parameterised as a four-level categorical variable with non-sex-dependent thresholds, anaemia demonstrated stepwise increases in attributable costs relative to patients without anaemia (Fig. 2). As a continuous variable, each 10 g L^−1^ increase in haemoglobin was associated with mean cost decrease of 1% (aRoM 0.99, 95% CI 0.98–0.99). As a nonlinear continuous restricted cubic spline, decreasing costs were associated with increasing haemoglobin concentration with an inflection point at 130 g L^−1^. This pattern was consistent at 90 and 365 days (Supplementary Appendix VIII). After adding estimated transfusion-attributable costs (Supplementary Appendix VII), the adjusted RoM was similar to the results of the primary analysis (aRoM 1.05, 95% CI 1.05–1.07) with mean absolute adjusted cost difference of $2879 (95% CI $2870–$2889). Accounting for survival time, anaemia was significantly associated with increased costs (RoM 1.03, 95% CI 1.02–1.04).

### Effect modification

**Fig 3:**
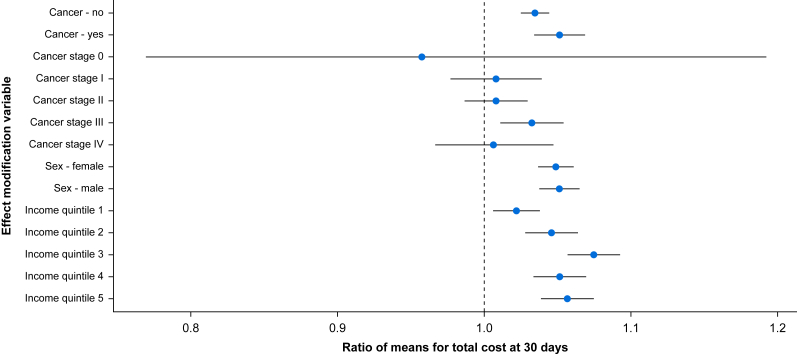
Forest plot of effect modification analyses for the association of anaemia and cost at 30 days. For each association, the figure reports the ratio of means (large circle) and 95% confidence interval comparing mean costs between those with *vs* without anaemia. Effect modifiers that were significant at the 5% level were cancer stage (among patients with cancer) and neighbourhood income quintile.

Figure 3 provides a summary of pre-specified binary and categorical effect modification analyses. No significant effect modification was identified by sex (*P*=0.77) or cancer status (*P=*0.12). Significant effect modification was identified by income quintiles (*P*=0.01), and among cancer patients by tumour stage (*P*<0.001). When continuous terms were analysed, those with greater frailty experienced a steady decrease in anaemia-attributable relative cost increase, which was statistically significant up to a frailty index score of 0.3 (Supplementary Appendix IXa). Although anaemic patients experienced significant cost increases across age range, the association peaked at age 55 years and demonstrated a nadir at age 75 years, with minimal difference in the RoM between the two ages at 30 days (Supplementary Appendix IXb). The interaction of continuous haemoglobin concentration with sex demonstrated that females had lower mean costs across haemoglobin concentration up to 166 g L^−1^ (Supplementary Appendix IXc).

### Clinical outcomes

Table 3 provides unadjusted and adjusted effect estimates for all secondary outcomes (outcome rates are reported in Supplementary Appendix X). Adjusted odds of transfusion and MAEs were greater for patients with anaemia (OR 4.34, 95% CI 4.04–4.66 and OR 1.14, 95% CI 1.03–1.27, respectively). Patients with anaemia experienced longer LOS (aRoM 1.08, 95% CI 1.07–1.10), and were more likely to be readmitted to hospital (adjusted OR 1.16, 95% CI 1.08–1.24). When anaemia was present before surgery, patients spent fewer days alive and at home (aRoM 0.95, 95% CI 0.95–0.96).

**Table 3:** Effect estimates for secondary outcomes with respect to preoperative anaemia status. CI, confidence interval; RBC, red blood cell. ∗Estimation unit reported as odds ratio. ^†^Estimation unit reported as ratio of means. ^‡^Adjusted covariates: age, sex, neighbourhood income quintile, rural *vs* urban residence, Elixhauser comorbidities, presence of colon or rectal cancer tissue diagnosis, frailty index score, preoperative healthcare resource utilisation, the specific surgical procedure, receipt of chemotherapy in the 6 months before surgery, estimated glomerular filtration rate, and presence of inflammatory bowel disease.

Outcome	Unadjusted relative effect measure (95% CI)	Adjusted^‡^ relative effect measure (95% CI)
RBC transfusion∗	5.41 (4.96–5.90)	4.34 (4.04–4.66)
In-hospital complications (composite)∗	2.34 (2.16–2.54)	1.14 (1.03–1.27)
Index length of hospital stay^†^	1.21 (1.19–1.22)	1.08 (1.07–1.10)
Readmissions∗	1.31 (1.23–1.39)	1.16 (1.08–1.24)
Days alive at home^†^	0.89 (0.88–0.90)	0.95 (0.95–0.96)

## Discussion

In a population-based, retrospective cohort study of adult patients undergoing elective colorectal surgery in Ontario, preoperative anaemia was significantly associated with an adjusted cost increase of $2672 CAD per person in the 30 days after surgery. This association was consistent across sensitivity analyses that involved differing assumptions and representations of preoperative haemoglobin concentration. Preoperative anaemia was also significantly associated with increased rates of adverse clinical outcomes, including transfusion, LOS, readmissions, and reduced days alive at home. These findings can inform the possible value and economic benefits of future efforts aimed at anaemia management.

Robust estimates of costs attributable to preoperative anaemia are lacking.^2^ Given the high prevalence, precise estimates of attributable costs are crucial to understand the impact of preoperative anaemia on resource-constrained healthcare systems and estimate the value of potentially expensive processes and interventions such as PBM programmes and i.v. iron therapies. To date, a single-centre study of 851 elective colorectal surgery patients estimated that preoperative anaemia was associated with an increase of $3027 CAD (95% CI $2664–$3397) in index hospitalisation costs.^2^ Our study estimated a similar degree of attributable costs over the first 30 postoperative days ($2671 CAD [95% CI $2661–$2680]) while adjusting for a more robust set of confounders and capturing all costs across a universal healthcare system. Cost differences continued to increase at 90 and 365 days, which could suggest that the clinical and subsequently, health system impacts of anaemia become more pronounced over time.^28^, ^29^, ^30^ However, interpretation of outcomes that are temporally farther away from the date of surgery and factor of study (i.e. preoperative anaemia) are limited.^31^

Anaemia in colorectal surgery is more prevalent with higher attributable costs than in other surgical populations including orthopaedic (12% prevalence, €426 or ∼$600 CAD in total joint arthroplasty) and gynaecologic (23% prevalence, $865 CAD in hysterectomy) surgery.^32^, ^33^, ^34^ Although hysterectomies are limited to biologically female individuals only, the observed higher costs in colorectal surgery do not appear to be related to sex differences, as our analyses did not identify sex as an effect modifier. Instead, the higher cost difference in colorectal surgery may be attributable to the inclusion of complex cancer cases and the greater physiologic stress of colorectal surgery leading to higher rates of postoperative complications.^35^ In our analyses, higher stage of cancer (stage 0–IV) was identified as an effect modifier among patients with cancer. In contrast, the presence of any cancer across all colorectal surgery patients was not. Although seemingly counterintuitive, this result likely reflects at least two factors. First, the analysis of cancer stage was limited to those in our cohort with a cancer diagnosis (i.e. a different risk set from the full population), and second, most cancers were less advanced, which might have diluted the effect of cancer in the full population.

Developing efficacious and cost-effective strategies to manage preoperative anaemia must remain a priority, especially given the population-level and societal impact of anaemia on the healthcare system. In Ontario, with a population of 14 million people, we estimate that anaemia contributed to more than $56 million in health system spending across the 10 yr of our study, or more than $5.5 million per year. As iron-deficiency anaemia secondary to gastrointestinal bleeding is a major aetiological factor for preoperative anaemia in colorectal surgery populations, strategies to address anaemia have typically included preoperative iron supplementation. Unfortunately, existing studies reveal complexities that may limit the effectiveness of this approach. Specifically, the multicentre PREVENTT trial involving 487 patient assessed the efficacy of a single i.v. iron therapy on anaemic patients undergoing major abdominal surgery. This study did not find evidence of reduced transfusion risk (RR 0.98, 95% CI 0.68–1.43) or adverse outcomes (RR 0.89, 95% CI 0.52–1.55). Nonetheless, substantial opportunities still exist to optimise anaemia treatment, informed in part by the PREVENTT trial.^12^ For example, although a pragmatic ‘one-size-fits-all’ approach may not be ideal, multidisciplinary PBM programmes that stress clinician awareness, differentiation of common causes, and institution of early therapy may be more effective in improving outcomes and reducing costs; however, appropriately powered randomised trials are still warranted.^2^^,^^7^^,^^8^^,^^36^

Our study features several strengths and limitations. Although our population-based design should support generalisability to similar populations and health systems, we cannot estimate generalisability to substantively different jurisdictions. In particular, for countries with much higher or lower absolute healthcare costs, our estimate of absolute attributable cost increase may not be relevant, and readers should prefer our relative estimates, while also considering the similarities and differences between delivery of surgical care in Canada and elsewhere. The internal validity of our findings should also be considered, including protocol pre-registration, use of validated measures of exposure, outcomes, and patient-level costing algorithms, and control for a robust set of confounders. Pre-planned sensitivity analyses supported the consistency and robustness of our findings, including concordance between our primary analysis using multiple imputation to account for missing exposure data and complete case analysis, suggesting that missing data were non-differential. It is also crucial that readers recognise that our study did not test or compare any interventions to treat or correct preoperative anaemia, so we are unable to draw any direct conclusions about cost-effectiveness. As the strength of attribution between an exposure measured before surgery and outcomes measured after surgery is expected to weaken over time, especially if unmeasured mediators or time-varying covariates emerge, interpretation of 90- and 365-day costs should be done with caution. Unfortunately, we could not identify the aetiology of anaemia, limiting our ability to explore aetiologic effect modification. Additionally, we used thresholds for anaemia according to the WHO definitions (i.e. <120 g L^−1^ in women and <130 g L^−1^ in men). Some experts suggest that haemoglobin <130 g L^−1^ should be considered anaemia, regardless of sex.^37^ As we used the WHO definitions, we might have captured more severe cases of anaemia in women leading to overestimated effects; had a single threshold for anaemia been used, our estimates might have been slightly different.^13^ However, in sensitivity analyses we found no effect modification by sex. Furthermore, the cost outcome was limited to the perspective of a publicly funded healthcare system in Ontario, Canada. Therefore, societal costs (i.e. loss of individual economic productivity, informal care costs from family members) and out-of-pocket expenses from patients (i.e. costs of travel) were not captured.

### Conclusions

In patients undergoing adult colorectal surgery, we estimate that the presence of anaemia before surgery is associated with increased costs of $2672 CAD (aROM 1.05, 95% CI 1.04–1.06), and significantly increased rates of adverse clinical and patient-centred outcomes in the 30 days after surgery. These data highlight the substantial impact of anaemia on colorectal surgery patients and the healthcare system. Efficacious and cost-effective interventions and processes are needed to inform management of perioperative anaemia, which remains prevalent in colorectal surgery patients.

## Authors’ contributions

All listed authors made substantial contributions to conception and/or design of the study and provided critical revisions for the pre-registered study protocol, statistical analysis plan, and submitted manuscript. All authors approve of the final version to be published and agree to be accountable for all aspects of the work.

Study design: LP, HM, TR, KT, JP, TZ, GM, PJD, RT, DM

Study conception: HM, TR, KT, JP, TZ, GM, PJD, DM

Data interpretation: LP, HM, KT, PJD, DM

Writing first draft of the manuscript: LP

Critical revision of the manuscript: HM, TR, KT, JP, TZ, GM, PJD, RT

Data analysis: RT

Guided writing of first draft of manuscript with multiple critical revisions: DM

## Acknowledgements

This study was supported by ICES, which is funded by an annual grant from the Ontario Ministry of Health (MOH) and the Ministry of Long-Term Care (MLTC). This document used data adapted from the Statistics Canada Postal CodeOM Conversion File, which is based on data licensed from Canada Post Corporation, and/or data adapted from the Ontario Ministry of Health Postal Code Conversion File, which contains data copied under license from ©Canada Post Corporation and Statistics Canada. Parts of this material are based on data and/or information compiled and provided by: MOH, Canadian Institute for Health Information. The analyses, conclusions, opinions, and statements expressed herein are solely those of the authors and do not reflect those of the funding or data sources; no endorsement is intended or should be inferred.

## Declaration of interest

DIM receives salary support from The Ottawa Hospital Department of Anesthesiology Alternate Funds Association, a Research Chair from the University of Ottawa Faculty of Medicine (Ottawa, ON, Canada), and the Mid-Career Knowledge Translation Fellowship from Physician Services Inc. Based on study questions PJD originated and grants he has written, he has received grants from Abbott Diagnostics, AOP, AstraZeneca, Bayer, Boehringer Ingelheim, Bristol-Myers-Squibb, Cloud DX, Coviden, Octapharma, Philips Healthcare, Roche Diagnostics, Roche, Siemens, Stryker, and Trimedic. He has also participated in advisory board meetings for GlaxoSmithKline, Bayer, Quidel Canada, Trimedic, an expert panel meeting with AstraZeneca, Boehringer Ingelheim, and Roche, and international meetings with AOP. He has also provided consultancy to Astra Zeneca. He is supported by the McMaster University/Hamilton Health Sciences Chair in Perioperative Care and a Tier 1 Canada Research Chair in Perioperative Care Research.

## Data access

The data used in this study were obtained from ICES. Those interested in accessing the data should contact ICES for permission.
