# Supplementary material for: Associations of preoperative anaemia with healthcare resource use and outcomes after colorectal surgery: a population-based cohort study
Source: Br J Anaesth. 2024 Apr 20;133(1):58–66. doi: 10.1016/j.bja.2024.03.018 (PMC11213985; doi:10.1016/j.bja.2024.03.018)
Supplement: Multimedia component 1 [file mmc1.docx]

**Supplementary file for “The association between preoperative anemia and healthcare resource use and outcomes after colorectal surgery: a population-based cohort study”**

**Supplementary Table 1:** Baseline characteristics of study population with and without hemoglobin value

| **Demographics** | **Hemolgobin Present N=54,286** | **Hemolgobin Absent**  **N=15,618** | **Absolute standardized difference**** |
| --- | --- | --- | --- |
| Age (median, range)) | 67 (57-76) | 65 (55-74) | 0.12 |
| Female | 26,573 (49.0%) | 7,460 (47.8%) | 0.12 |
| Rural | 7,852 (14.5%) | 2,463 (15.8%) | 0.04 |
| Neighborhood Income Quintile |  |  |  |
| 1 (highest) | 10,387 (19.1%) | 2,983 (19.1%) | 0 |
| 2 | 10,914 (20.1%) | 3,232 (20.7%) | 0.01 |
| 3 | 10,856 (20.0%) | 3,163 (20.3%) | 0.01 |
| 4 | 10,977 (20.2%) | 3,142 (20.1%) | 0 |
| 5 (lowest) | 11,018 (20.3%) | 3,048 (19.5%) | 0.02 |
| Academic Hospital (vs. Community) | 17,837 (32.9%) | 4,760 (30.5%) | 0.05 |
| **Baseline health status** |  |  |  |
| Frailty Index (mean ± SD) | 0.12 ± 0.07 | 0.11 ± 0.07 | 0.12 |
| Hemoglobin value (mean ± SD) | 126.94 ± 20.87 | NA | NA |
| Days from haemoglobin test to surgery (mean ± SD) | 17.90 ± 19.93 | NA | NA |
| eGFR (mean ± SD) | 79.27 ± 20.95 | 76.84 ± 19.99 | 0.12 |
| IBD | 2,167 (4.0%) | 528 (3.4%) | 0.03 |
| Elixhauser comorbidities |  |  |  |
| Diabetes complicated | 4,806 (8.9%) | 1,038 (6.6%) | 0.08 |
| Diabetes uncompicated | 8,970 (16.5%) | 2,141 (13.7%) | 0.08 |
| Congestive heart failure | 1,846 (3.4%) | 405 (2.6%) | 0.05 |
| Hypertension uncomplicated | 12,431 (22.9%) | 3,538 (22.7%) | 0.01 |
| Hypertension complicated | 114 (0.2%) | 30 (0.2%) | 0 |
| Chronic pulmonary disease | 2,399 (4.4%) | 848 (5.4%) | 0.05 |
| Dementia | 290 (0.5%) | 75 (0.5%) | 0.01 |
| Cerebrovascular disease | 747 (1.4%) | 182 (1.2%) | 0.02 |
| Chronic renal disease | 648 (1.2%) | 131 (0.8%) | 0.04 |
| Dialysis | 214 (0.4%) | 51 (0.3%) | 0.01 |
| Cancer | 39,350 (72.5%) | 9,678 (62.0%) | 0.23 |
| Cancer with metastases | 9,156 (16.9%) | 2,096 (13.4%) | 0.1 |
| Peripheral vascular disease | 798 (1.5%) | 189 (1.2%) | 0.02 |
| Liver disease | 462 (0.9%) | 84 (0.5%) | 0.04 |
| Peptic ulcer disease | 908 (1.7%) | 203 (1.3%) | 0.03 |
| Rheumatic disease | 193 (0.4%) | 52 (0.3%) | 0 |
| Hemiparesis or hemiplegia | 124 (0.2%) | 35 (0.2%) | 0 |
| Atrial fibrillation | 2,039 (3.8%) | 456 (2.9%) | 0.05 |
| Venous thromboembolism | 325 (0.6%) | 74 (0.5%) | 0.02 |
| Cardiac valve disease | 767 (1.4%) | 181 (1.2%) | 0.02 |
| Disease of the pulmonary circulation | 886 (1.6%) | 211 (1.4%) | 0.02 |
| Coagulopathy | 584 (1.1%) | 165 (1.1%) | 0 |
| Obesity | 1,796 (3.3%) | 542 (3.5%) | 0.01 |
| Weight loss | 1,313 (2.4%) | 294 (1.9%) | 0.04 |
| Blood loss anaemia | 4,270 (7.9%) | 1,202 (7.7%) | 0.01 |
| Nutritional deficiency anaemia | 407 (0.7%) | 85 (0.5%) | 0.03 |
| Alcohol abuse | 561 (1.0%) | 202 (1.3%) | 0.02 |
| Drug abuse | 151 (0.3%) | 66 (0.4%) | 0.02 |
| Psychoses | 116 (0.2%) | 47 (0.3%) | 0.02 |
| Depression | 722 (1.3%) | 251 (1.6%) | 0.02 |
| **Baseline cancer status** |  |  |  |
| Cancer diagnosis | 35,108 (64.7%) | 9,569 (61.3%) | 0.07 |
| Colon cancer | 21,047 (38.8%) | 5,344 (34.2%) | 0.09 |
| Rectal cancer | 10,218 (18.8%) | 3,343 (21.4%) | 0.06 |
| Cancer stage (among cancer patients) | 30,046 (55.3%) | 7,737 (49.5%) | 0.12 |
| 0 | 92 (0.2%) | 41 (0.3%) | 0.02 |
| I | 6,498 (12.0%) | 2,275 (14.6%) | 0.08 |
| II | 7,778 (14.3%) | 2,275 (14.6%) | 0.01 |
| III | 8,209 (15.1%) | 2,805 (18.0%) | 0.08 |
| IV | 1,663 (3.1%) | 485 (3.1%) | 0 |
| Previous chemotherapy | 5,935 (10.9%) | 1,477 (9.5%) | 0.05 |
| **Surgery type** |  |  |  |
| Partial Colectomy | 35,135 (64.7%) | 9,877 (63.2%) | 0.03 |
| Subtotal Colectomy | 588 (1.1%) | 159 (1.0%) | 0.01 |
| Proctocolectomy | 261 (0.5%) | 42 (0.3%) | 0.03 |
| Low anterior resection | 13,377 (24.6%) | 3,964 (25.4%) | 0.02 |
| Abdominoperineal resection | 4,925 (9.1%) | 1,576 (10.1%) | 0.03 |

Standard Deviation [SD]; estimated Glomerular Filtration Rate [eGFR]; Inflammatory Bowel Disease [IBD]

**Appendix I:** STROBE and RECORD Checklist

STROBE Statement—Checklist of items that should be included in reports of ***cohort studies***

|  | Item No | Recommendation |
| --- | --- | --- |
| **Title and abstract** | 1 | (*a*) Indicate the study’s design with a commonly used term in the title or the abstract  -Provided |
|  |  | (*b*) Provide in the abstract an informative and balanced summary of what was done and what was found  -Page 3-4 |
| Introduction | | |
| Background/rationale | 2 | Explain the scientific background and rationale for the investigation being reported  -Page 5-6 |
| Objectives | 3 | State specific objectives, including any prespecified hypotheses  -Page 6, *introduction last paragraph* |
| Methods | | |
| Study design | 4 | Present key elements of study design early in the paper  -Page 6, *methods 1^st^ paragraph* |
| Setting | 5 | Describe the setting, locations, and relevant dates, including periods of recruitment, exposure, follow-up, and data collection  - Page 7 |
| Participants | 6 | (*a*) Give the eligibility criteria, and the sources and methods of selection of participants. Describe methods of follow-up  -Page 6-7 *Cohort* |
|  |  | (*b*) For matched studies, give matching criteria and number of exposed and unexposed  -N/A |
| Variables | 7 | Clearly define all outcomes, exposures, predictors, potential confounders, and effect modifiers. Give diagnostic criteria, if applicable  -Page 7 *Exposure*  -Page 7 *Outcomes*  -Page 8 *Confounders* and *Appendix II&VII* |
| Data sources/ measurement | 8* | For each variable of interest, give sources of data and details of methods of assessment (measurement). Describe comparability of assessment methods if there is more than one group  - Pages 7-8, 10 |
| Bias | 9 | Describe any efforts to address potential sources of bias  -Pages 8-10 |
| Study size | 10 | Explain how the study size was arrived at  -Page 8 (population-based analysis) |
| Quantitative variables | 11 | Explain how quantitative variables were handled in the analyses. If applicable, describe which groupings were chosen and why  - Page 9-11 |
| Statistical methods | 12 | (*a*) Describe all statistical methods, including those used to control for confounding  -Page 9-11 |
|  |  | (*b*) Describe any methods used to examine subgroups and interactions  -Page 9-10 |
|  |  | (*c*) Explain how missing data were addressed  -Page 9 |
|  |  | (*d*) If applicable, explain how loss to follow-up was addressed  -N/A |
|  |  | (*e*) Describe any sensitivity analyses  -Page 9-10 *Sensitivity analyses* |
| Results | | |
| Participants | 13* | (a) Report numbers of individuals at each stage of study—eg numbers potentially eligible, examined for eligibility, confirmed eligible, included in the study, completing follow-up, and analysed  -Page 10, *Results* *1^st^ paragraph* |
|  |  | (b) Give reasons for non-participation at each stage  -N/A |
|  |  | (c) Consider use of a flow diagram  -N/A |
| Descriptive data | 14* | (a) Give characteristics of study participants (eg demographic, clinical, social) and information on exposures and potential confounders  -Table 1, Page 10 *Results 1^st^ paragraph* |
|  |  | (b) Indicate number of participants with missing data for each variable of interest  -Page 9 |
|  |  | (c) Summarise follow-up time (eg, average and total amount)  - Page 7-8 |
| Outcome data | 15* | Report numbers of outcome events or summary measures over time  -Table 2, Page 10-12 |
| Main results | 16 | (*a*) Give unadjusted estimates and, if applicable, confounder-adjusted estimates and their precision (eg, 95% confidence interval). Make clear which confounders were adjusted for and why they were included  - Table 2-3, Page 8, 13, 14 |
|  |  | (*b*) Report category boundaries when continuous variables were categorized  -Present |
|  |  | (*c*) If relevant, consider translating estimates of relative risk into absolute risk for a meaningful time period  -N/A |
| Other analyses | 17 | Report other analyses done—eg analyses of subgroups and interactions, and sensitivity analyses  -Page 9-12 |
| Discussion | | |
| Key results | 18 | Summarise key results with reference to study objectives  -Page 13 *Discussion 1^st^ paragraph* |
| Limitations | 19 | Discuss limitations of the study, taking into account sources of potential bias or imprecision. Discuss both direction and magnitude of any potential bias  -Page 16 *Strengths and limitations* |
| Interpretation | 20 | Give a cautious overall interpretation of results considering objectives, limitations, multiplicity of analyses, results from similar studies, and other relevant evidence  -Page 15-16 |
| Generalisability | 21 | Discuss the generalisability (external validity) of the study results  -Page 14-16 |
| Other information | | |
| Funding | 22 | Give the source of funding and the role of the funders for the present study and, if applicable, for the original study on which the present article is based  -Present |

*Give information separately for exposed and unexposed groups.

**Unique RECORD checklist items**

RECORD 1.1: The type of data used should be specified in the title or abstract. When possible, the name of the databases used should be included.

-Please see Design section of abstract

RECORD 1.2: If applicable, the geographic region and timeframe within which the study took place should be reported in the title or abstract.

- Please see last Setting and Participant section in abstract

RECORD 1.3: If linkage between databases was conducted for the study, this should be clearly stated in the title or abstract.

- Please see Design section of abstract

RECORD 6.1: The methods of study population selection (such as codes or algorithms used to identify subjects) should be listed in detail. If this is not possible, an explanation should be provided.

- Please see Appendix for validated codes used to identify surgeries

RECORD 6.2: Any validation studies of the codes or algorithms used to select the population should be referenced. If validation was conducted for this study and not published elsewhere, detailed methods and results should be provided.

-For procedural codes please see reference 15

RECORD 6.3: If the study involved linkage of databases, consider use of a flow diagram or other graphical display to demonstrate the data linkage process, including the number of individuals with linked data at each stage.

-Because all linkages were complete we did not add a flow diagram.

RECORD 7.1: A complete list of codes and algorithms used to classify exposures, outcomes, confounders, and effect modifiers should be provided. If these cannot be reported, an explanation should be provided.

-Please see Appendix for complete list of codes

RECORD 12.1: Authors should describe the extent to which the investigators had access to the database population used to create the study population.

-Please see methods, where analyst access is described

RECORD 12.2: Authors should provide information on the data cleaning methods used in the study.

-Please see methods. Data used were part of regularly collected ICES data, therefore the analyst who cut the data did not need to do any data cleaning.

RECORD 12.3: State whether the study included person-level, institutional-level, or other data linkage across two or more databases. The methods of linkage and methods of linkage quality evaluation should be provided.

-Please see description of patient-level deterministic data linkage in Methods section.

RECORD 13.1: Describe in detail the selection of the persons included in the study (*i.e.,* study population selection) including filtering based on data quality, data availability and linkage. The selection of included persons can be described in the text and/or by means of the study flow diagram.

-Please see the *Cohort* section of the methods

RECORD 19.1: Discuss the implications of using data that were not created or collected to answer the specific research question(s). Include discussion of misclassification bias, unmeasured confounding, missing data, and changing eligibility over time, as they pertain to the study being reported.

-Please see limitations section.

RECORD 22.1: Authors should provide information on how to access any supplemental information such as the study protocol, raw data, or programming code.

-Due to privacy restriction is our provincial health administrative data we are unable to provide access to analytic data sets or programming code.

**Appendix II:** Canadian Classification of Intervention (CCI) codes for colon and rectal resections

| **CCI Code** | **CCI description** | **Clinical description** |
| --- | --- | --- |
| 1NM87 | Excision partial, large intestine | Hemicolectomy |
| 1NM89 | Excision total, large intestine | Subtotal Colectomy |
| 1NM91 | Excision radical, large intestine | Proctocolectomy |
| 1NQ87 | Excision partial, rectum | Low anterior resection |
| 1NQ89 | Excision total, rectum | Abdominoperineal resection |

**Appendix III: Healthcare resource utilization included in cost outcome**

Cost analyses followed previously established methods. These methods account for direct and indirect costs, the latter of which are estimated based on patient-level resource-intensity weights and a patient’s location in their trajectory across their perioperative journey. The following healthcare resource costs, as outlined by Wodchis *et al.*^1^, were included in our cost outcomes:

- inpatient hospitalizations,
- ambulatory care (i.e., emergency room, urgent care clinics),
- inpatient rehabilitation,
- complex continuing care,
- long term care,
- inpatient mental health,
- home care services (i.e., visiting health professionals including nurses, physiotherapists, occupational therapists, social workers, speech-language pathologists, dieticians; personal care and support services; homemaking services; community health services),
- all physician services (including outpatient visits, interpretation of laboratory exams and diagnostic tests)
- all prescription drugs, and
- assistive devices

**Appendix IV:** Definitions of covariates included in model

**Length of stay:** Total number of days that a patient remained in hospital throughout index admission, starting from day of admission as day 0 until day of discharge, derived from Canadian Institutes for Health Information Discharge Abstract Database (DAD).

**Days alive at home:** a patient-centered outcome calculated as the number of days alive in the 30 days after surgery minus any days in hospital (DAD), emergency department (National Ambulatory Care Reporting System), or institutional care (National Rehabilitation Reporting System).

**Readmissions within 30-days of discharge:** Number of times a patient is readmitted to hospital care within 30 days of discharge from index admission (Discharge Abstract Database).

**In-hospital major adverse cardiovascular events (MACE):** composite of myocardial infarction [MI], acute kidney injury [AKI], atrial arrhythmia, ventral arrhythmia, heart failure, ischemic stroke, transient ischemic attack [TIA], in-hospital death based on type 2 (arising during hospitalization) diagnostics codes from the Discharge Abstract Database based on International Classification of Diseases (ICD), Tenth Edition codes.

**Myocardial infarction:** DAD – DXCODE(i)=I21, I22, I20, I23.82, I24 with DXTYPE(i)=2

**Acute kidney injury:** DAD – DXCODE(i) =N17 with DXTYPE(i)=2

**Atrial arrhythmia:** DAD – DXCODE(i)=I48, I495 with DXTYPE(i)=2

**Ventricular arrhythmia:** DAD – DXCODE(i)=I490, with DXTYPE(i)=2

**Heart failure:** DAD - DXCODE(i)=I50, with DXTYPE(i)=2

**Ischemic stroke:** DAD - DXCODE(i)= I63, I64 with DXTYPE(i)=2

**Transient ischemic attack:** DAD - DXCODE(i)= G45 with DXTYPE(i)=2

**In-hospital death:** Discharge status listed as dead from index hospitalization.

**Receipt of any red blood cell (RBC) transfusion:** DAD- BTREDBC=’Yes’

**Appendix V:** Covariates adjusted for in analyses

All models were adjusted for covariates as follows: age (restricted cubic spline with 3 knots), sex (binary), income quintile (categorical), procedure (categorical), rurality (binary), Elixhauser comorbidities (each as a binary variable), year of surgery (restricted cubic spline with 3 knots), frailty (continuous), total health system costs accrued in the year before surgery (restricted cubic spline with 3 knots), perioperative chemotherapy (binary, within 6 months of surgery), inflammatory bowel disease (binary), and an indicator for surgical oncology status (non-cancer, cancer[rectal-Stage I-II], cancer[rectal-Stage III-IV], cancer[colon-Stage I-II] cancer[colon-Stage III-IV]) and eGFR (restricted cubic spline with 3 knots). All adjusted models accounted for clustering by hospital using generalized estimating equation methods. Of note, preoperative healthcare resource was total health system costs accrued in the year prior to surgery. This was adjusted for because greater preoperative healthcare use has been found to be associated with greater post-operative healthcare use.^3,4^ Since prior patterns of healthcare utilization can impact post-operative care utilization, we adjusted for this variable as a potential confounder.

**Appendix VIa: Diagnostics for multiple imputation**

Missing values were only present for the anemia exposure status (22.3%) and eGFR (4.3%). Our multiple imputation model included all covariates included in our adjusted models, as well as all primary and secondary outcomes. We created 25 imputed data sets using PROC MI (SAS v 9.4 for Windows, SAS Institute, Cary, NC, USA) via multiple imputation by chained equations under fully conditional specification.^2^ Predictive mean matching was used to impute missing values. Analyses were then run as specified in the primary analysis, and combined using PROC MIANALYZE. Relative imputation efficiency for the exposure variable was 99.9%.

**Appendix VIb:** Table of observed, imputed, and total population hemoglobin values by 25 imputed data sets

|  | **Imputation Number** | | | | | | | | | | | | |
| --- | --- | --- | --- | --- | --- | --- | --- | --- | --- | --- | --- | --- | --- |
|  | **1** | **2** | **3** | **4** | **5** | **6** | **7** | **8** | **9** | **10** | **11** | **12** | **13** |
| **Test Present-Complete Case** | | | | | | | | | | | | | |
| **Hgb Mean** | 126.94 | 126.94 | 126.94 | 126.94 | 126.94 | 126.94 | 126.94 | 126.94 | 126.94 | 126.94 | 126.94 | 126.94 | 126.94 |
| **Standard deviation** | 20.87 | 20.87 | 20.87 | 20.87 | 20.87 | 20.87 | 20.87 | 20.87 | 20.87 | 20.87 | 20.87 | 20.87 | 20.87 |
| **Test Absent-Imputed** | | | | | | | | | | | | | |
| **Hgb Mean** | 128.78 | 128.95 | 129.22 | 129.09 | 128.93 | 129.24 | 129.28 | 128.62 | 129.01 | 128.87 | 129.11 | 128.84 | 128.76 |
| **Standard deviation** | 20.67 | 20.61 | 20.78 | 20.62 | 20.65 | 20.74 | 20.53 | 20.85 | 20.55 | 20.59 | 20.50 | 20.66 | 20.65 |
| **Total Population** | | | | | | | | | | | | | |
| **Hgb Mean** | 127.35 | 127.39 | 127.45 | 127.42 | 127.38 | 127.45 | 127.46 | 127.31 | 127.40 | 127.37 | 127.42 | 127.36 | 127.34 |
| **Standard deviation** | 20.84 | 20.83 | 20.87 | 20.84 | 20.84 | 20.87 | 20.82 | 20.88 | 20.82 | 20.83 | 20.81 | 20.84 | 20.84 |
| **Imputation number**  **(continued)** | **14** | **15** | **16** | **17** | **18** | **19** | **20** | **21** | **22** | **23** | **24** | **25** |  |
| **Test Present-Complete Case** | | | | | | | | | | | | | |
| **Hgb Mean** | 126.94 | 126.94 | 126.94 | 126.94 | 126.94 | 126.94 | 126.94 | 126.94 | 126.94 | 126.94 | 126.94 | 126.94 |  |
| **Standard deviation** | 20.87 | 20.87 | 20.87 | 20.87 | 20.87 | 20.87 | 20.87 | 20.87 | 20.87 | 20.87 | 20.87 | 20.87 |  |
| **Test Absent-Imputed** | | | | | | | | | | | | | |
| **Hgb Mean** | 128.61 | 128.93 | 129.06 | 128.85 | 129.02 | 128.88 | 128.97 | 128.84 | 128.80 | 128.81 | 128.95 | 129.02 |  |
| **Standard deviation** | 20.69 | 20.45 | 20.39 | 20.70 | 20.26 | 20.46 | 20.61 | 20.72 | 20.60 | 20.62 | 20.56 | 20.62 |  |
| **Total Population** | | | | | | | | | | | | | |
| **Hgb Mean** | 127.31 | 127.38 | 127.41 | 127.36 | 127.40 | 127.37 | 127.39 | 127.36 | 127.35 | 127.36 | 127.39 | 127.40 |  |
| **Standard deviation** | 20.84 | 20.80 | 20.79 | 20.85 | 20.76 | 20.80 | 20.83 | 20.86 | 20.83 | 20.83 | 20.82 | 20.84 |  |

**Hgb=hemolgobin in g/L**

**Appendix VIc: Distribution of 25 stratified complete cases and imputed cases**


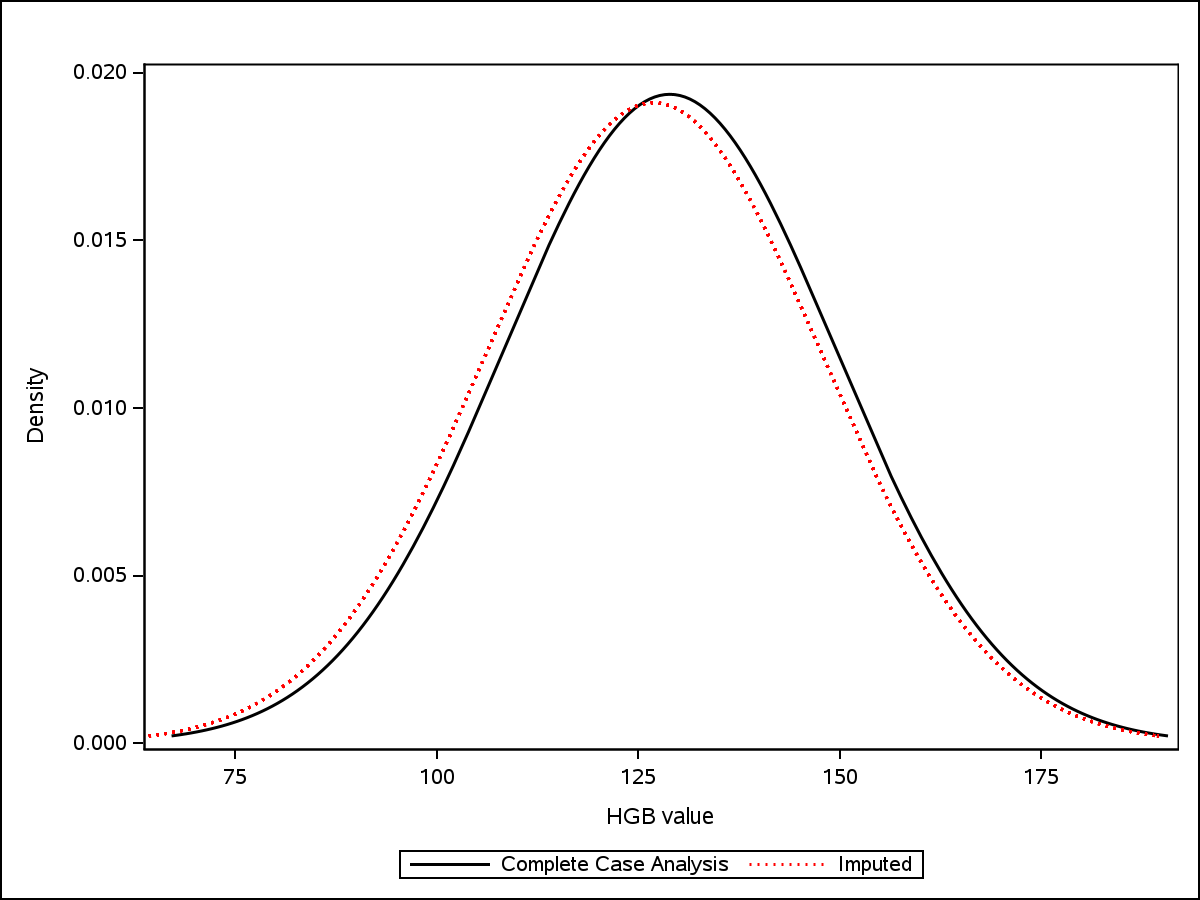


***this is a visual representation of Appendix Vb**

**Appendix VII:** Description of post-hoc transfusion-adjusted analysis:

For this analysis we used an estimate of $666 in 2014 CAD per 1 unit produced (i.e., donor vein to hospital costs), which was adjusted for inflation to $805 in 2022 CAD.^5^ As our data did not capture the number of units transfused (only a binary indicator of RBC transfusion receipt), we identified from the literature that among colorectal surgery patients who receive a transfusion, the average number of units transfused is 1.5.^6,7^ This allowed us to add an additional $1,207 CAD to the health system costs of each transfused patient (regardless of anemia status). Therefore, for the sensitivity analysis the the dependent variable in our primary adjusted model was total measured health system cost plus the estimated costs of red blood cell unit production, which was used to re-estimate a transfusion adjusted attributable cost.

**Appendix VIII:** Sensitivity analysis utilizing non-linear restricted cubic spline to explore association of continuous hemoglobin levels and (A) 30- (B) 90- and (B) 365- day costs.

1. 30-Day Costs
2. 90-Day Costs
3. 365-Day Costs

**Appendix IX:** Effect modifier analyses using interaction terms of continuous hemoglobin with (A) Frailty, (B) Age, and (C) Sex at 30-, 90-, and 365-days following surgery

1. Frailty


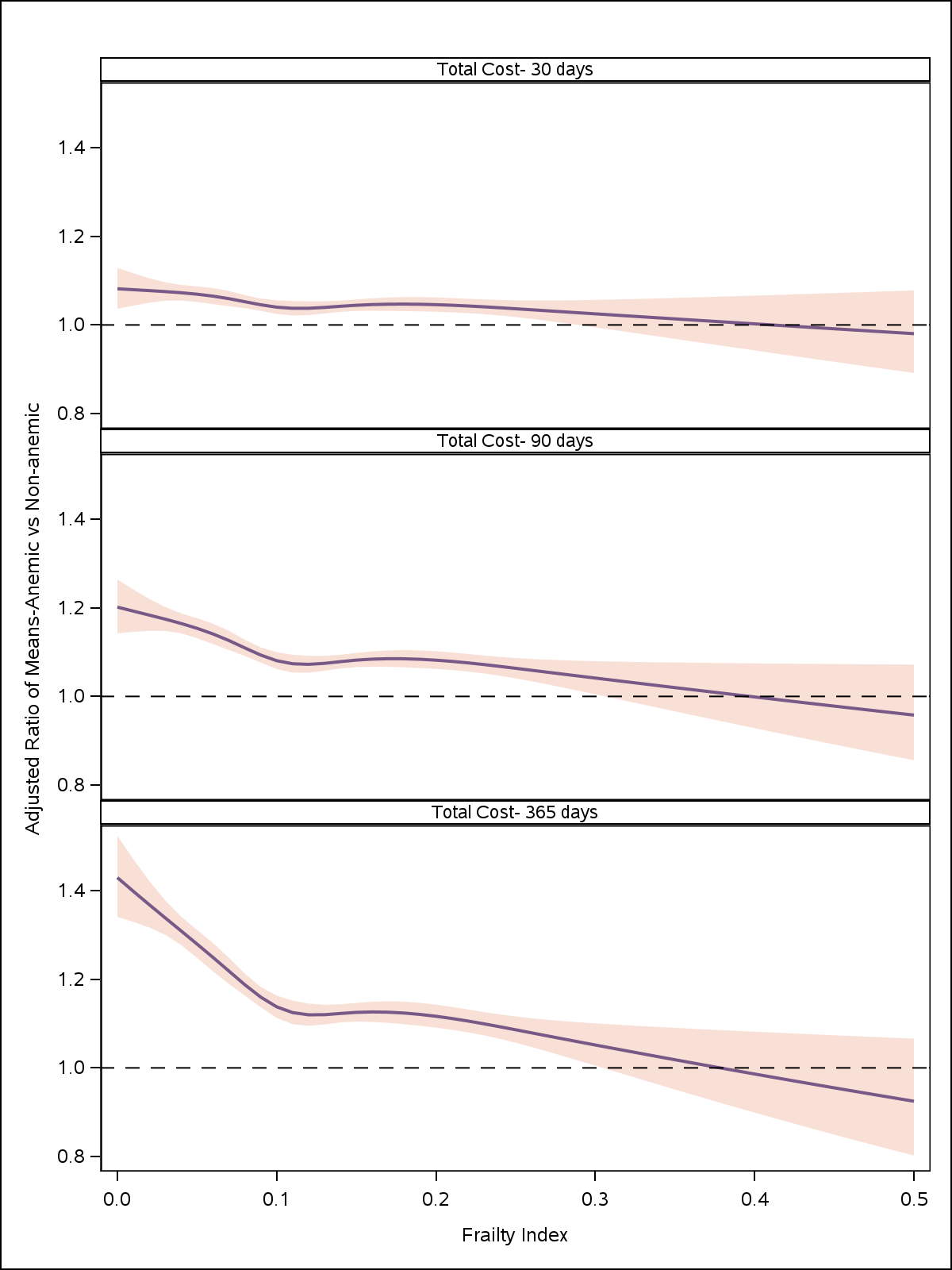


1. Age


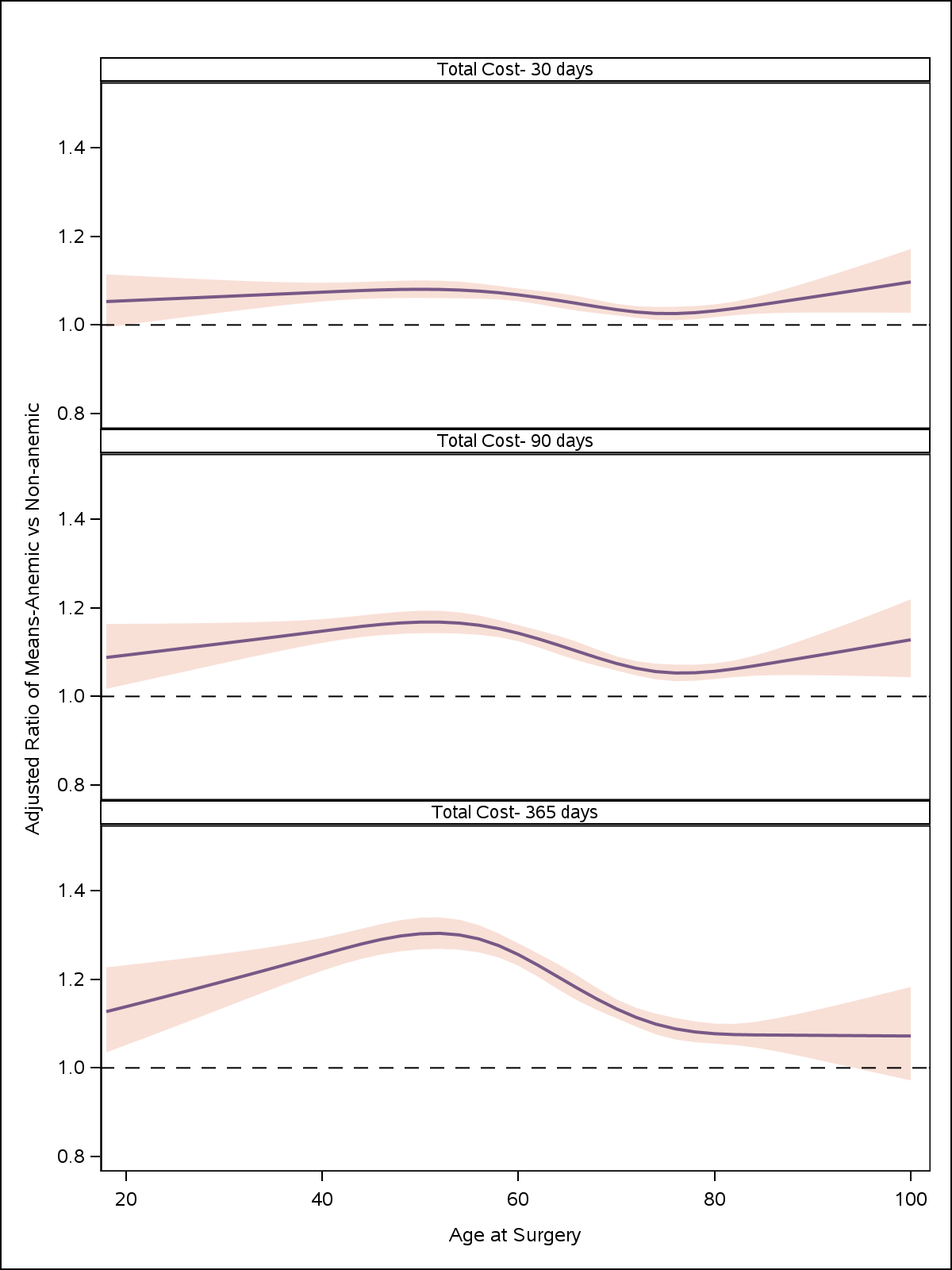


1. Sex


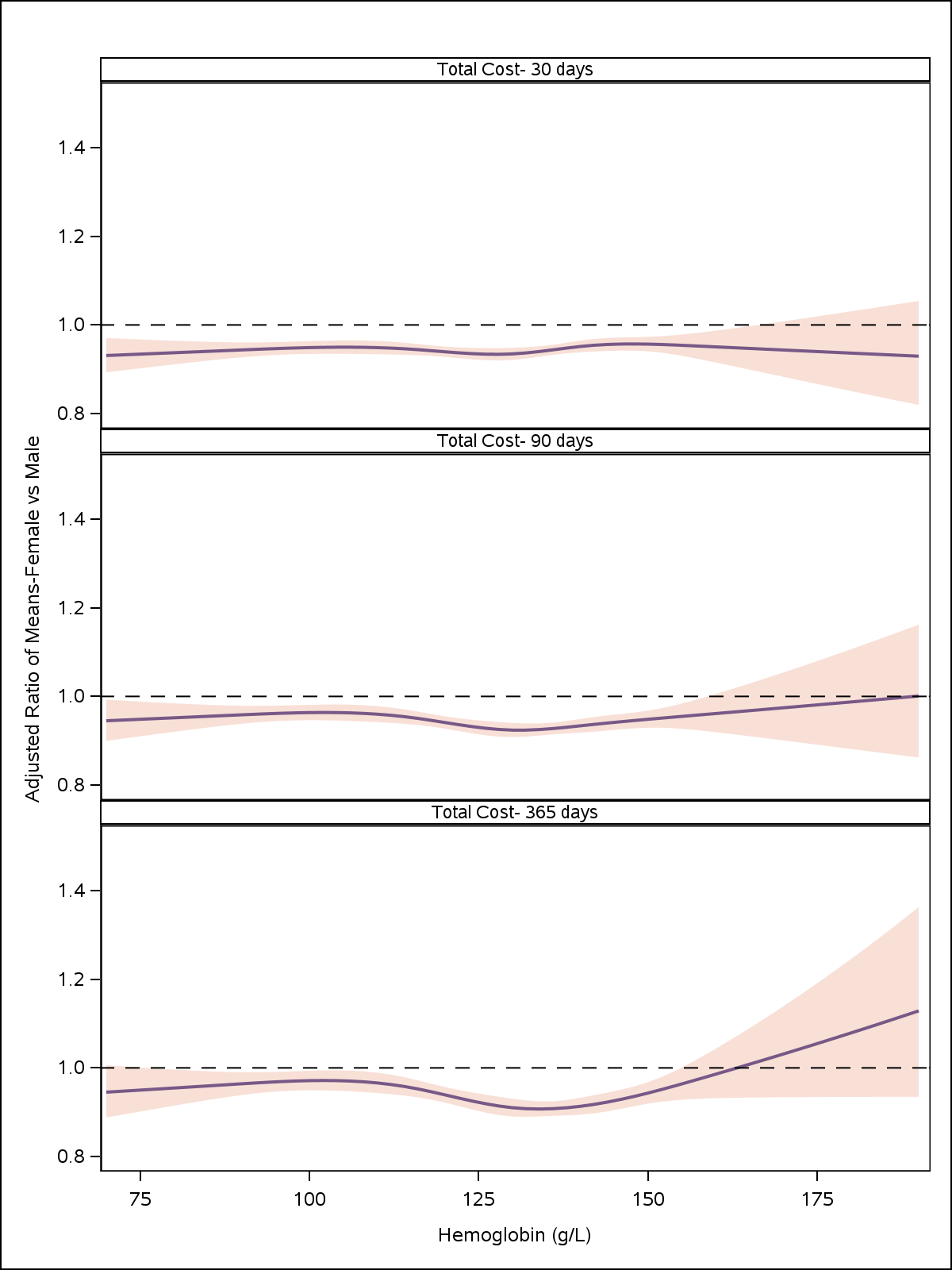


**Appendix X:** Crude Secondary outcomes with respect to preoperative anemia status

|  | **Anemic** | | |
| --- | --- | --- | --- |
| **Outcome** | **No**  **(N=33,022)** | **Yes**  **(N=21,264)** | **Absolute standard Difference** |
| RBC Transfusion | 1,931 (5.8%) | 5,205 (24.5%) | 0.54 |
| In-hospital MACE (composite) | 952 (2.9%) | 1,393 (6.6%) | 0.17 |
| MI | 130 (0.4%) | 241 (1.1%) | 0.09 |
| AKI | 198 (0.6%) | 301 (1.4%) | 0.08 |
| Atrial arrhythmia | 430 (1.3%) | 456 (2.1%) | 0.06 |
| Ventricular arrhythmia | <=5 (0.0%) | <=5 (0.0%) | 0.01 |
| Heart Failure | 103 (0.3%) | 232 (1.1%) | 0.09 |
| Ischemic stroke | 38 (0.1%) | 56 (0.3%) | 0.03 |
| TIA | <=5 (0.0%) | 9 (0.0%) | 0.02 |
| In-hospital death | 258 (0.8%) | 545 (2.6%) | 0.14 |
| Index length of hospital stay* | 7.4 ± 11.8 | 9.7 ± 14.9 | 0.17 |
| Readmissions | 2,505 (7.6%) | 2,075 (9.8%) | 0.08 |
| Days alive at home* | 21.85 ± 6.14 | 19.45 ± 8.04 | 0.33 |

All Outcomes reported as N (%) unless otherwise indicated; Red Blood Cell [RBC]; myocardial infarction [MI]; acute kidney injury [AKI]; atrial arrhythmia; ventral arrhythmia; heart failure; ischemic stroke; transient ischemic attack [TIA]; in-hospital death; Complex Continuing Care [CCC]; major adverse cardiovascular events [MACE]

*Outcome reported in days ± standard deviation

**References**

1. Wodchis W, Bushmeneva K, Nikitovic M, McKillop I. Guidelines on Person-Level Costing Using Administrative Databases in Ontario. Health System Performance Research Network [Internet]. 2013; Available from: http:// www.hsprn.ca/uploads/files/Guidelines_on_PersonLevel_ Costing_May_2013.pdf. A

2. Giganti MJ, Shepherd BE. Multiple-Imputation Variance Estimation in Studies With Missing or Misclassified Inclusion Criteria. Am J Epidemiol. 2020 Jul 20;189(12):1628–32.

3. Clewley D, Rhon DI, Flynn TW, Sissel CD, Cook CE. Does Health Care Utilization Before Hip Arthroscopy Predict Health Care Utilization After Surgery in the US Military Health System? An Investigation Into Health-Seeking Behavior. Journal of Orthopaedic & Sports Physical Therapy. 2018 Nov;48(11):878–86.

4. Creager A, Kleven AD, Kesimoglu ZN, Middleton AH, Holub MN, Bozdag S, et al. The Impact of Preoperative Healthcare Utilization on Complications, Readmissions, and Postoperative Healthcare Utilization Following Total Joint Arthroplasty. J Arthroplasty. 2022 Mar;37(3):414–8.

5. Lagerquist O, Poseluzny D, Werstiuk G, Slomp J, Maier M, Nahirniak S, et al. The cost of transfusing a unit of red blood cells: a costing model for Canadian hospital use. ISBT Science Series. 2017;12(3):375–80.

6. Benoist S, Panis Y, Pannegeon V, Alves A, Valleur P. Predictive factors for perioperative blood transfusions in rectal resection for cancer: A multivariate analysis of a group of 212 patients. Surgery. 2001 Apr;129(4):433–9.

7. Shaker EH, Fayek ES, Elrawas MM. Evaluation of efficacy and safety of a single dose Tranexamic acid in reducing blood loss during colorectal cancer surgery. A randomised, placebo controlled, double-blinded study. Indian J Anaesth. 2023 Feb;67(2):194–200.
